# Acid Catalyzed Formation of C–C and C–S Bonds via Excited State Proton Transfer

**DOI:** 10.3390/molecules24071318

**Published:** 2019-04-03

**Authors:** Alessandro Strada, Mattia Fredditori, Giuseppe Zanoni, Stefano Protti

**Affiliations:** 1 Department of Chemistry, University of Pavia, Viale Taramelli 10, 27100 Pavia, Italy; alessandro.strada02@universitadipavia.it; 2 PhotoGreen Lab, Department of Chemistry, University of Pavia, Viale Taramelli 10, 27100 Pavia, Italy; mattia.fredditori01@universitadipavia.it

**Keywords:** excited state proton transfer, acid catalyzed synthesis, photocatalysis, tris(bipyridine)ruthenium(II) chloride

## Abstract

The behavior of 2-naphthol and 7-bromo-2-naphthol as organic photoacids are exploited in organic synthesis for the preparation of benzyl sulfides (using a trichloroacetimidate derivative as the starting substrate) and polycyclic amines via acid catalyzed condensation of 1,2,3,4-tetrahydroisoquinoline with aldehydes.

## 1. Introduction

**Scheme 1:**
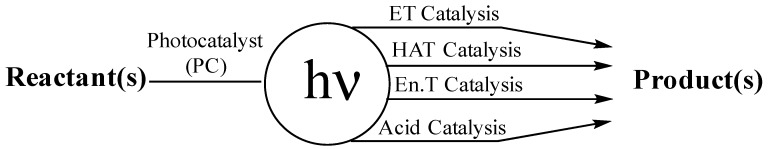
Photocatalytic Activation of Substrates.

Photocatalysis emerged in the last decade as an established strategy for organic synthesis [1,2,3,4,5], in view of the versatility of the approach and the mild conditions adopted, including working at room temperature, the adoption of aqueous solvents and the use of photons as a traceless reagent [6,7]. In the photocatalytic approach, the absorption of a photon by the compound that acts as the photocatalyst (PC in Scheme 1) leads to dramatic changes that can impact different aspects of its reactivity. As an example, irradiation of a molecule can affect its redox properties by increasing the oxidation or the reduction potential, in turn opening the way to an electron transfer (ET) step. Such behavior is the pillar of the visible-light photoredox catalytic approach, the synthetic potential of which has been widely demonstrated, starting from the seminal works of Mac Millan [8,9] and Yoon [10].

In some cases, the excited photocatalyst is able to activate selectively a C–H bond of a reactant via a direct Hydrogen Atom Transfer (HAT) path, to generate the corresponding carbon centered radical that can be trapped in turn by a suitable olefin, leading to the formation of a new C–C bond [11]. However, direct HAT processes are currently limited to few (photo)catalysts, namely aromatic ketones [12] and some polyoxometalate derivatives (e.g., decatungstate salts) [13]. In some cases, activation of the substrate towards the desired reaction occurs via a simple energy transfer (also known as sensitization) from the excited catalyst [14,15].

A further approach relies on the electron density shift occurring when a compound is promoted to the corresponding excited state, which can result in a dramatic increase of the acidity, by down to 13 pKa units [16,17,18,19]. A wide range of organic molecules (including substituted phenols and naphthols) exhibit such a property, and are therefore defined as “organophotoacids”.

Whereas HAT, sensitization and especially photoredox catalysis found an impressive range of applications in synthesis, little attention has been given to the use of organophotoacids, which, in in most cases, were mainly used in deprotection reactions [20] as well as in the protection of alcohols as tetrahydropyran ethers [21]. However, interest in such an approach recently emerged. Lei et al. described the potentialities of the xanthene dye Eosin Y [22,23] in the visible light driven acetalization of aldehydes; the same organophotoacid was recently employed in the stereoselective preparation of 2-deoxyglycosides [24]. Toshima et al. exploited the decrease of the *p*Ka value (down to five magnitude orders) of a chiral thiourea derivative in photoinduced stereo-controlled glycosylation reactions [25]. A significant contribution to the use of organophotoacids in synthesis has been offered by Hanson et al., who optimized the visible-light enantioselective protonation of silyl enol ethers, using an Ir(III) complex as the photosensitizer, 7-bromo-2-naphthol as the photocatalyst and an excess of phenol as the proton source [26]. The approach was recently implemented, and the use of a chiral organophotoacid was able to induce some degree of enantioselectivity [27].

## 2. Results and Discussion

In view of these recent results, we reasoned that organophotoacids could potentially play a role in a wide range of synthetic processes, including C–S and C–C bond formation. With this target in mind, we embarked on the exploratory study of a model reaction, namely the acid catalyzed nucleophilic substitution occurring on trichloroacetimidate derivative **1** [28,29]. The process was investigated by using different light sources and, on the basis of the available literature, 2-naphthol (**PA1**) and 7-bromo-2-naphthol (**PA2**) were considered in the role of organophotoacid candidates. The choice of the solvent was carried out on the basis of their compatibility with the reaction conditions, which precluded the use of alcoholic solvents. The reaction was monitored by GC and the product yield was measured by calibration curve obtained by comparison with an authentic sample. The obtained results are summarized in Table 1.

Irradiation of **1** (0.10 M) at 366 nm, in the presence of an excess of **2** (0.50 M) and 2-naphthol (**PA1**, 30 mol%) as the photoacid in DMSO did not afford the desired thiolation, and **1** underwent exclusively thermal decomposition to the corresponding benzyl 2,2,2-trichloroacetate (entry 1). However, small amounts of **3** (up to 21% yield) were observed when moving from apolar (*n*-hexane and toluene, entries 2,3) to polar media, such as dichloroethane, diethyl ether and acetonitrile (entries 4–6). Better results were obtained when moving to dichloromethane, where **3** was formed in 28% yield (entry 7). The efficiency of the process was further improved by increasing either the equivalent of **2** (up to 1.0 M, entry 8) or of photoacid (up to 40% yield when **PA1** was used in 50 mol%, entry 10). Irradiation with different wavelength (310 nm, 15 W, Hg lamp), afforded expected thiolation product with a comparable yield (entry 11). Similar results were obtained when using 7-bromo-2-naphthol (**PA2**) as the organophotoacid, with the benzyl sulfide **3** obtained in up to 47% yield (entries 12, 13). As in the case of **PA1**, the reaction yield dropped when acetonitrile was used as the solvent (entry 14). Finally, the reaction took place only to a minor extent in the absence of the photoacid and of light, respectively (entries 15,16).

With this promising results in hand, we decided to investigate the potentialities of organophotoacid catalysis in the preparation of alkaloids **6a**,**b**, from tetrahydroisoquinoline **5** and salicylaldehydes **4a**,**b** (in turn obtained by adapting a known synthesis procedure; see Supplementary Materials for further details) [30]. Unfortunately, the conditions optimized for the synthesis of **3** were found inefficient to achieve this target (Table 2, entry 1). Taking inspiration from the work of Hanson et al. [26], we decided to move to visible light irradiation by adopting Ru(bpy)_3_^2+^ as the photosensitizer. In this case, due to the poor solubility of the complex in DCM, experiments were performed in MeCN, and light emitting diodes (LEDs) were used as the light source. Gratifyingly, a discrete amount of **6a** (19% yield, entry 2) was obtained when irradiating a solution of **4a** and a slight excess of tetrahydroisoquinoline **5** (1.2 equiv) in the presence of 2-naphthol (50 mol%) and Ru(bpy)_3_^2+^ (5 mol%) as the photoacid and the photocatalyst, respectively. A change of the **4a:5** stoichiometric ratio from 1:1.2 to 1.5:1 (entries 3,4) led to a significant improvement of the reaction efficiency, and the desired alkaloid **6a** was isolated in up to 65% yield. Further increase in the amount of **4a** did not significantly influence the process (entry 5), whereas in the absence of **PA1** the product was obtained in low yield, due to the unsatisfactory consumption of **5** (entry 6). Good results were also obtained with **PA2**. In this case, irradiation of a Ru^II^-sensitized solution of **4a** and **5** in dry acetonitrile in the presence of 7-bromo-2-naphthol as the photoacid and 1 equiv of phenol afforded **6a** a 62% yield (entry 7). Finally, the protocol was extended to the preparation of methoxy substituted derivative **6b**. In this case, however, the desired product was obtained only in modest yield (25%, entry 8).

**Scheme 2:**
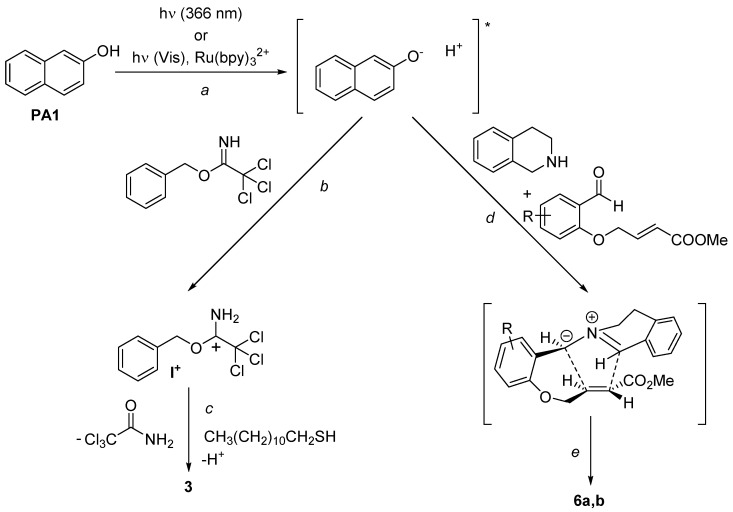
Proposed mechanism for the acid induced formation of sulfides **3** and alkaloids **6a**,**b** via photoexcited 2-napthol (* PA1).

As stated in the Introduction, the behavior of naphthol derivatives as organophotoacids has been widely investigated [31,32,33]. In particular, the pKa* value for the photoexcited 2-naphthol (**PA1**) in water has been calculated as 2.8, with an enhancement of more than six orders of magnitude with respect to the value for the ground state (pKa = 9.45) [34]. Analogous results have been described in the literature for 7-bromo-2-naphthol, for which a ΔpKa > 10 was observed upon excitation [26]. In this case, intersystem crossing from the singlet excited state to the triplet of **PA2** is favored by the presence of a bromine atom as substituent, thus the **^3^PA2** state is responsible for the photoacidity of the molecule [35]. Such excited states could also be populated by using Ru^II^(bpy)_3_Cl_2_ as the photosensitizer under visible light irradiation. For the sake of simplicity, the mechanism of the reactions has been described in Scheme 2 for the case of 2-naphthol. Thus, irradiation of 2-naphthol results in the formation of a short-lived, photoexcited acid, that can interact with the proton sensitive moiety of the substrate. As depicted in Scheme 2, interaction of the released proton with trichloroacetimidates (path b) results in the formation of the cationic intermediate **I^+^**, which undergoes nucleophilic substitution to form the desired sulfide **3** [36]. On the other hand, the photostimulated acidity of **PA1** and **PA2** is also responsible for the formation of azomethine ylides **7** via proton-induced coupling (path d) of tetrahydroquinoline **5** and aldehydes **4a**,**b** [30]. As also described in literature [26,27] phenol (1 equiv) could act as a sacrificial proton source to restore the starting photoacid. The intermediate then afforded the desired alkaloids **6a**,**b** (path e).

In conclusion, we demonstrated that molecules exhibiting a behavior as organophotoacids can be efficiently used in organic synthesis, in both C–S and C–C bond formation processes, avoiding the use of aggressive acids as well as of harsh conditions.

## 3. Material and Methods

### 3.1. General

All the chemicals and solvents used for the synthesis of **3**, **4a**,**b** and **6a**,**b** were commercially available and used as received. The solvents used for the irradiations (GC or HPLC grade purity), as well as 2-naphthol, 7-bromo-2-naphthol and tris(2,2’-bipyrydyl)dichlororuthenium(II) hexahydrate were likewise commercially available and used as received. Dichloromethane (GC grade purity) and Toluene (GC grade purity) were purified by distillation over the drying agent CaCl_2_. All sensitive reactions were carried out under a positive static atmosphere of Ar. Syringes, needles and the other glassware were dried at 140 °C for at least one night and allowed to cool in a desiccator over P_2_O_5_ before use. Routine monitoring of reactions was performed using silica gel 60 mesh (0.25 mm) aluminum-supported Thin Layer Chromatography (TLC) plates (purchased from Merck, Rome, Italy). Compounds were visualized by UV irradiation at a wavelength of 254 nm or stained by exposure to a 0.5% solution of vanillin in H_2_SO_4_/EtOH, followed by charring. NMR spectra were recorded at 200 or 300 (for ^1^H) and 75 MHz (for ^13^C), respectively, in the solvents indicated; chemical shifts (δ) are given in ppm relative to TMS, and coupling constants (*J*) are in hertz (Hz). The solvent signals were used as references, and the chemical shifts were converted to the TMS scale (CDCl_3_: δ-C 77.00; residual CHCl_3_ in CDCl_3_: 7.26 ppm). The number of H-atoms attached to each C-atom was determined by DEPT experiments. Photochemical reactions were carried out by using argon purged solutions in quartz tubes. Irradiations were performed in a multi-lamp reactor fitted with 10 × 15 W phosphor-coated Hg lamps (emission centered at 366 or 310 nm). In selected cases, the processes were also performed by irradiation in Pyrex vials in a reactor equipped with LED 1 W (emission centered at 410 nm). For compounds **3**, **4a** and **6a**, GC/MS analyses were carried out using a single quadrupole GC/MS system. The GC oven temperature was held at 80 °C for 1 min, increased to 280 °C by a temperature ramp of 10 °C min^-1^ and held for 5 min. For compounds **4b** and **6b**, the GC oven temperature was held at 80 °C for 1 min, increased to 280 °C by a temperature ramp of 10 °C min^−1^ and held for 15 min. Mass spectral analysis was carried out in full scan mode.

### 3.2. Analytical Data for Compounds **3**–**6**

#### 3.2.1. Synthesis of Benzyl(dodecyl)sulfide (**3**)

Compound **3** was prepared by following a known procedure [37]. Spectroscopical data were in accordance with the literature [38]. ^1^H-NMR (200 MHz, CDCl_3_) δ(ppm); 7.40–7.15 (m, 5H), 3.72 (s, 2H), 2.42 (t, *J* = 7.3 Hz, 2H), 1.55 (d, *J* = 9.0 Hz, 4H), 1.26 (s, 24H), 0.99–0.77 (m, 4H). GC–MS (*m*/*z*): 90.9 (100), 92.0 (32).

#### 3.2.2. Photochemical synthesis of **3**

A nitrogen-saturated solution of benzyl trichloroacetimidate (**1**, 0.1 M), dodecylsulfide (2, 0.5 M, 5 equiv), and 7-bromo-2-naphthol (0.05 M, **PA2**, 50 mol%) and Ru[(bpy)_3_]^2+^ (5 mol%) in dry MeCN (1 mL) was irradiated for 12 h in a multi-lamp reactor equipped with 10 phosphor coated Hg lamps (15 W, λ_em_ = 366 nm). The product formed (**3**) was identified and quantified by comparison with calibration curves of authentic samples.

#### 3.2.3. Synthesis of (*E*)-methyl 4-(2-formylphenoxy)but-2-enoate (**4a**)

Compound **4a** was prepared by following a known procedure [30]. ^1^H-NMR (300 MHz, CDCl_3_) δ(ppm): 10.56 (s, 1H), 7.87 (ddd, *J* = 7.7, 1.8, 0.4 Hz, 1H), 7.56 (ddd, *J* = 8.5, 7.4, 1.9 Hz, 1H), 7.19–7.03 (m, 3H), 6.95 (d, *J* = 8.5 Hz, 0H), 6.24 (dt, *J* = 15.8, 2.1 Hz, 1H), 4.88–4.81 (m, 2H), 3.79 (s, 3H). ^13^C NMR (75 MHz, CDCl_3_) δ(ppm): 189.1, 166.1, 160.0, 141.4, 135.8, 128.7, 125.0, 121.9, 121.3, 112.4, 66.7, 51.7

#### 3.2.4. Synthesis of Methyl-6a,7,7a,12,13,14a-hexahydro-6H chromeno[3′,4′:4,5]pyrrolo[2,1-a]isoquinoline-7-carboxylate (**6a**)

Compound **6a** was prepared by following a known procedure [30]. ^1^H-NMR (300 MHz, CDCl_3_) δ(ppm): 7.51–7.42 (m, 1H), 7.25–7.13 (m, 1H), 7.15–7.04 (m, 3H), 7.06–6.91 (m, 2H), 6.88 (dd, *J* = 8.2, 1.3 Hz, 1H), 4.48 (dd, *J* = 8.1, 3.6 Hz, 2H), 4.34 (dd, *J* = 11.5, 3.9 Hz, 1H), 4.23 (dd, *J* = 11.5, 2.7 Hz, 1H), 3.59 (dd, *J* = 9.3, 6.2 Hz, 1H), 3.49 (ddd, *J* = 11.3, 8.6, 4.4 Hz, 1H), 3.30 (s, 1H), 3.08–2.88 (m, 3H). ^13^C-NMR (75 MHz, CDCl_3_) δ(ppm): 174.4, 155.5, 135.1, 133.4, 130.4, 128.4, 128.3, 127.4, 126.3, 125.1, 121.2, 116.6, 65.9, 62.4, 62.3, 51.5, 51.3, 46.0, 40.5, 28.6. GC-MS (*m*/*z*): 27.9 (78), 76.9 (100), 114.9 (90), 130.9 (78), 144.9 (90), 203.0 (54).

#### 3.2.5. Synthesis of (*E*)-methyl 4-(2-formyl-5-methoxyphenoxy)but-2-enoate **4b**

Compound **4b** was prepared by following a known procedure [30]. ^1^H-NMR (300 MHz, CDCl_3_) δ(ppm) 10.37 (s, 1H), 7.84 (d, *J* = 8.7 Hz, 1H), 7.11 (dt, *J* = 15.7, 4.0 Hz, 1H), 6.59 (ddd, *J* = 8.6, 2.3, 0.8 Hz, 1H), 6.40 (d, *J* = 2.2 Hz, 1H), 6.23 (dt, *J* = 15.7, 2.0 Hz, 1H), 4.80 (dd, *J* = 4.1, 2.1 Hz, 2H), 3.87 (s, 3H), 3.78 (s, 3H). ^13^C-NMR (75 MHz, CDCl_3_) δ(ppm): 187.7, 166.1, 165.9, 161,7, 141.2, 130.8, 121.9, 119.1, 106.2, 98.9, 66.7, 55.6, 51.7.

#### 3.2.6. Synthesis of Methyl-methoxy-6a,7,7a,12,13,14a-hexahydro-6H-chromeno[3′,4′:4,5]pyrrolo[2,1-a]isoquinoline-7-carboxylate **6b**

Compound **6b** was prepared by following a known procedure [30]. ^1^H-NMR (300 MHz, CDCl_3_) δ(ppm): 7.35 (d, *J* = 8.5 Hz, 1H), 7.13–7.05 (m, 3H), 7.04–6.98 (m, 1H), 6.56 (dd, *J* = 8.6, 2.6 Hz, 1H), 6.42 (d, *J* = 2.6 Hz, 1H), 4.45 (d, *J* = 7.1 Hz, 2H), 4.33 (dd, *J* = 11.5, 3.9 Hz, 1H), 4.21 (dd, *J* = 11.5, 2.8 Hz, 1H), 3.79 (s, 3H), 3.57 (dd, *J* = 9.3, 6.1 Hz, 1H), 3.45 (q, *J* = 8.0, 5.9 Hz, 1H), 3.29 (s, 3H), 2.96 (qd, *J* = 12.8, 11.4, 5.8 Hz, 4H). ^13^C-NMR (75 MHz, CDCl_3_) δ(ppm): 174.3, 159.8, 156.4, 135.1, 130.8, 128.3, 127.4, 126.3, 125.1, 108.4, 101.1, 66.1, 62.4, 61.9, 55.2, 51.5, 51.3, 45.9, 40.5, 28.5. GC-MS (*m*/*z*): 161.1 (100), 175.1 (90), 203.1 (62), 204.1 (48), 235.1 (100), 266.7 (30)

#### 3.2.7. Photochemical synthesis of **6a,b**

A nitrogen-saturated solution of 1,2,3,4-tetrahydroisoquinoline **5** (0.1 M), salicylaldehydes **4a**,**b** (0.15 M, 1.5 equiv), 2-naphthol (0.075 M, **PA1**, 50 mol% with respect to the chosen salicylaldehyde) and Ru[(bpy)_3_]^2+^ (5 mol%) in dry MeCN (1 mL, the reaction was carried out in the presence of 4 Å MS) was poured in a Pyrex vial and irradiated at 410 nm for 24 h (LED, 1 W). The use of phenol (1 equiv) as the proton source in selected cases was also investigated. The product formed was identified and quantified by comparison with calibration curves of authentic samples.

## Figures and Tables

**Table 1:** Acid catalyzed synthesis of benzylsulfide **3**.

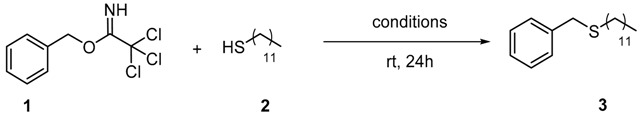			
Entry	Conditions ^a^	Light Source (nm)	Yield ^b^ (%)
1	DMSO, **1** (0.1 M), **2** (0.5 M), **PA1** (30 mol%)	366	-
2	Hexane, **1** (0.1 M), **2** (0.5 M), **PA1** (30 mol%)	366	13
3	PhMe, **1** (0.1 M), **2** (0.5 M), **PA1** (30 mol%)	366	15
4	DCE, **1** (0.1 M), **2** (0.5 M), **PA1** (30 mol%)	366	21
5	Et_2_O, **1** (0.1 M), **2** (0.5 M), **PA1** (30 mol%)	366	8
6	MeCN, **1** (0.1 M), **2** (0.5 M), **PA1** (30 mol%)	366	4
7	DCM, **1** (0.1 M), **2** (0.5 M), **PA1** (30 mol%)	366	28
8	DCM, **1** (0.1 M), **2** (1.0 M), **PA1** (30 mol%)	366	40
9	DCM, **1** (0.1 M), **2** (0.2 M), **PA1** (30 mol%)	366	12
10	DCM, **1** (0.1 M), **2** (0.5 M), **PA1** (50 mol%)	366	40
11	DCM, **1** (0.1 M), **2** (0.5 M), **PA1** (50 mol%)	310	21
12	DCM, **1** (0.1 M), **2** (0.5 M), PA**2** (30 mol%)	366	32
13	DCM, **1** (0.1 M), **2** (0.5 M), PA**2** (50 mol%)	366	47
14	MeCN, **1** (0.1 M), **2** (0.5 M), PA**2** (50 mol%) ^c^	366	9
15	DCM, **1** (0.1 M), **2** (0.5 M)	366	12
16	DCM, **1** (0.1 M), **2** (0.5 M), PA**2** (50 mol%) ^d^	- ^d^	-

^a^ mol% of 2-naphthol (**PA1**) and 7-bromo-2-naphthol (**PA2**) were calculated with respect to the amount of **1**; ^b^ Determined by GC analyses; ^c^ 1.5 mol% of Ru^II^ was used; ^d^ No light.

**Table 2:** Acid induced synthesis of alkaloids **6a**,**b**.

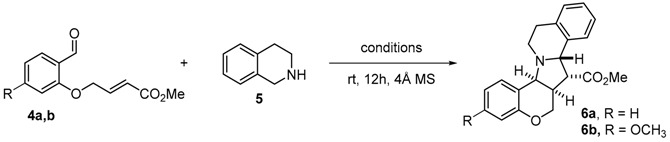			
Entry	Conditions ^a^	Light Source (nm)	Product (% Yield) ^b^
1	**4a** (0.1 M) **5** (0.12 M), **PA1** or **PA2** (50 mol%)	366	**6a**, < 5%
	dry DCM		
2	**4a** (0.1 M), **5** (0.12 M), **PA1** (50 mol%), Ru[(bpy)_3_]^2+^	410	**6a**, 19%
	(5 mol%), dry MeCN		
3	**4a** (0.1 M), **5** (0.1 M), **PA1** (50 mol%), Ru[(bpy)_3_]^2+^	410	**6a**, 43%
	(5 mol%), dry MeCN		
4	**4a** (0.15 M), **5** (0.10 M), **PA1** (50 mol%), Ru[(bpy)_3_]^2+^	410	**6a**, 65%
	(5 mol%), dry MeCN		
5	**4a** (0.2 M), **5** (0.10 M), **PA1** (50 mol%), Ru[(bpy)_3_]^2+^	410	**6a**, 63%
	(5 mol%), dry MeCN ^c^		
6	**4a** (0.15 M), **5** (0.10 M), Ru[(bpy)_3_]^2+^	410	**6a**, 20%
	(5 mol%), dry MeCN		
7	**4a** (0.15 M), **5** (0.10 M), **PA2** (30 mol%), Ru[(bpy)_3_]^2+^	410	**6a**, 62%
	(5 mol%), dry MeCN		
8	**4b** (0.15 M), **5** (0.10 M), **PA1** (50 mol%), Ru[(bpy)_3_]^2+^	410	**6b**, 25%
	(5 mol%), dry MeCN		

^a^ mol% of photocatalyst and **PA1**, **PA2** were calculated with respect to the amount of **1**; ^b^ Determined by GC analyses; ^c^ 1.0 equiv of phenol was used.

## Supplementary Materials

The following are available online at https://www.mdpi.com/1420-3049/24/7/1318/s1: Copy of the 1H and 13 NMR spectra of the synthesized compounds (Section S1), and UV-Visible spectra of compounds **PA1**, **PA2**, **1** and **4a** Figures S1–S4.
