# Supplementary material for: Acid Catalyzed Formation of C–C and C–S Bonds via Excited State Proton Transfer
Source: Molecules. 2019 Apr 3;24(7):1318. doi: 10.3390/molecules24071318 (PMC6480163; doi:10.3390/molecules24071318)
Supplement: Supplementary file 1 [file molecules-24-01318-s001.pdf]

## Supplementary information

# Acid catalyzed formation of C-C and C-S bonds via Excited State Proton Transfer

Alessandro Strada,[1] Mattia Fredditori,[1,2] Giuseppe Zanoni,*[1] Stefano Protti[2]*

*1 Department of Chemistry, University of Pavia, Viale Taramelli, 10-27100 Pavia, Italy*

*2 PhotoGreen Lab, Department of Chemistry, University of Pavia, Viale Taramelli 10, 27100 Pavia. Correspondence: stefano.protti@unipv.it; gz@unipv.it*

**Table of Contents.**

**(9 PAGES)**

1. $^{1}$H and $^{13}$C-NMR spectra of the synthesized compounds S2

2. UV-Visible spectra of compounds **PA1**,**PA2**, **1** and **4a**. S7

# 1. $^{1}H$ and $^{13}C$-NMR spectra of the synthesized compounds.

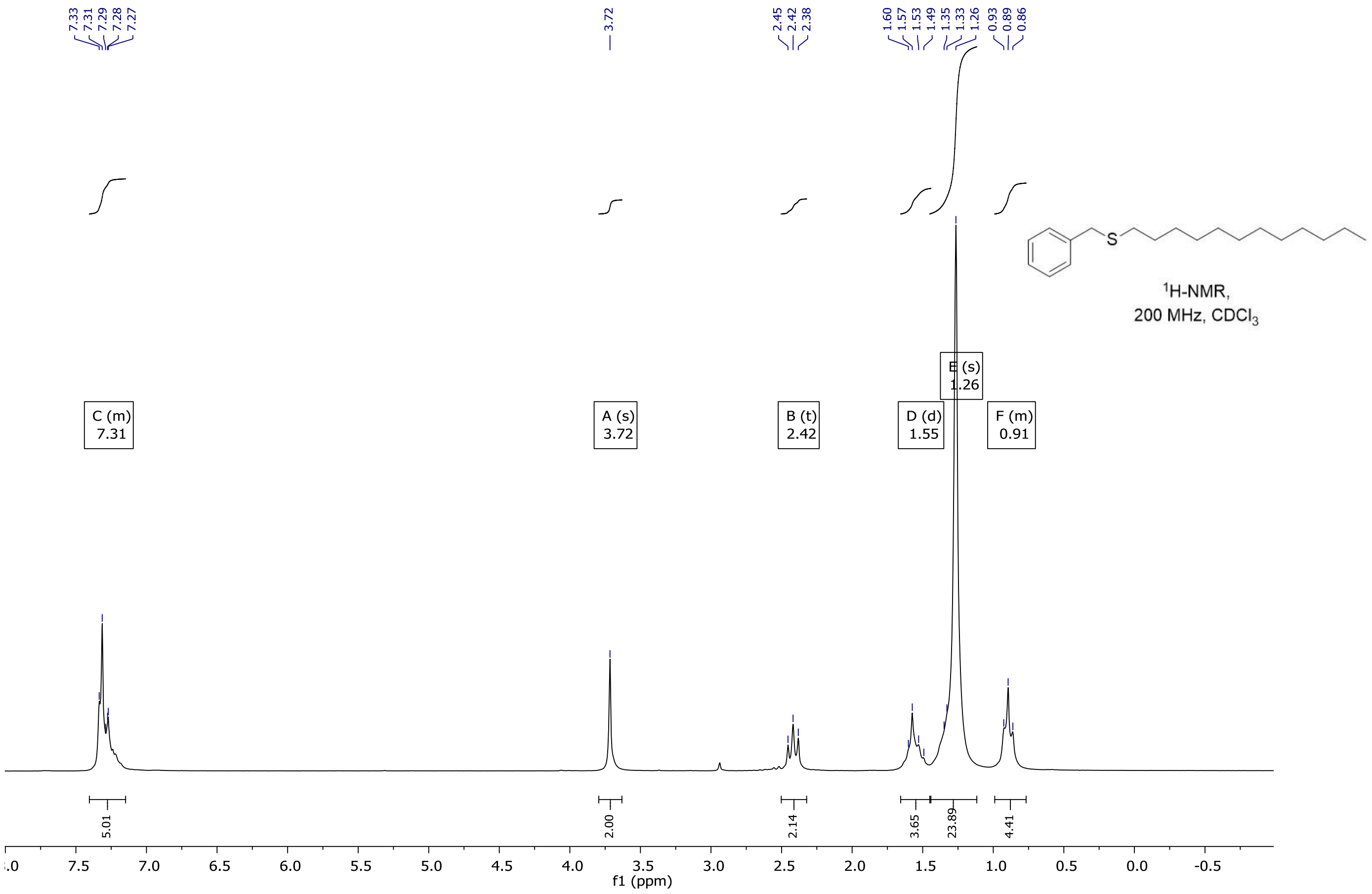

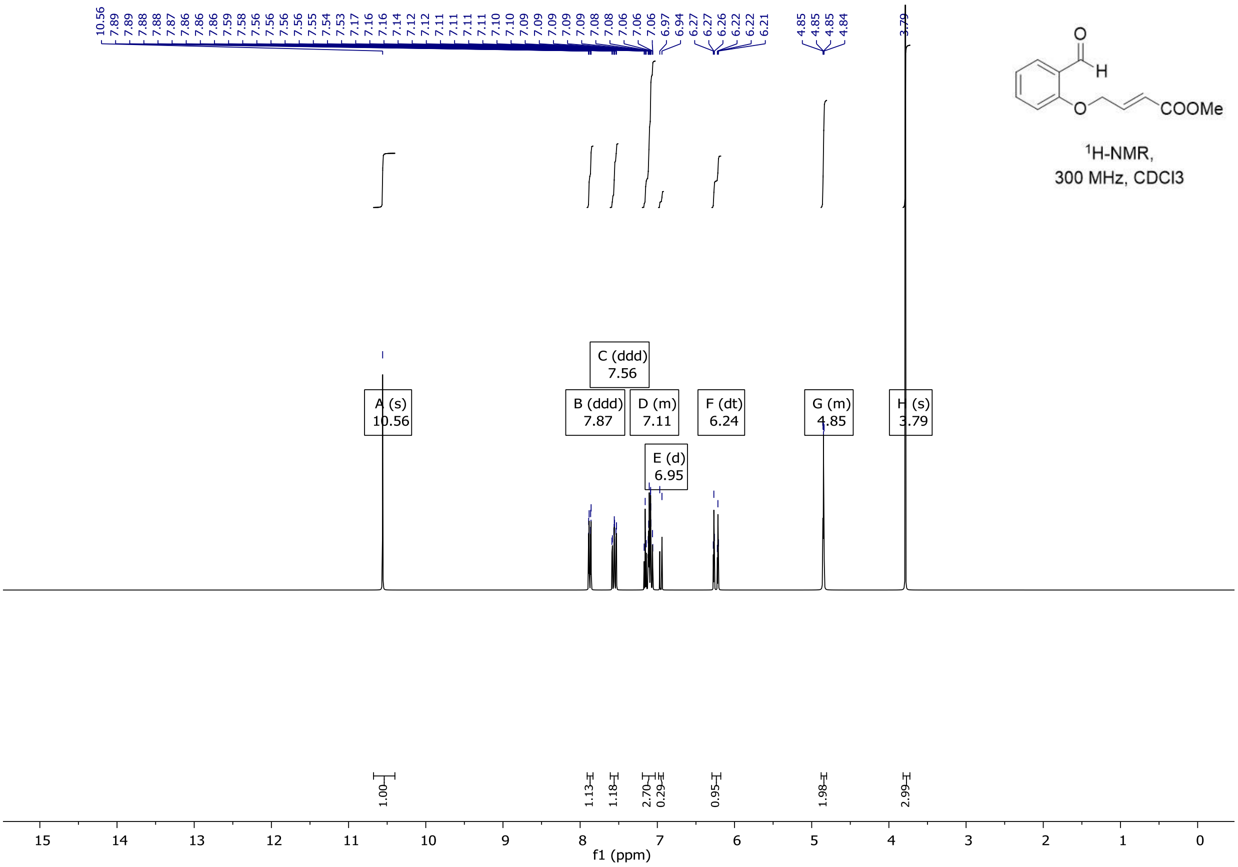
10.56
7.89
7.89
7.88
7.87
7.86
7.86
7.59
7.58
7.56
7.56
7.56
7.55
7.54
7.53
7.17
7.16
7.16
7.14
7.12
7.12
7.11
7.11
7.11
7.11
7.10
7.09
7.09
7.09
7.09
7.08
7.06
7.06
6.97
6.94
6.27
6.26
6.22
6.22
6.21
4.85
4.85
4.85
4.84
3.79
O
H
O
COOMe
$^{1}$H-NMR,
300 MHz, CDCl3
C (ddd)
7.56
A (s)
10.56
B (ddd)
7.87
D (m)
7.11
F (dt)
6.24
G (m)
4.85
H (s)
3.79
E (d)
6.95
1.00
1.13
1.18
2.70
0.29
0.95
1.98
2.99
15 14 13 12 11 10 9 8 7 6 5 4 3 2 1 0
f1 (ppm)

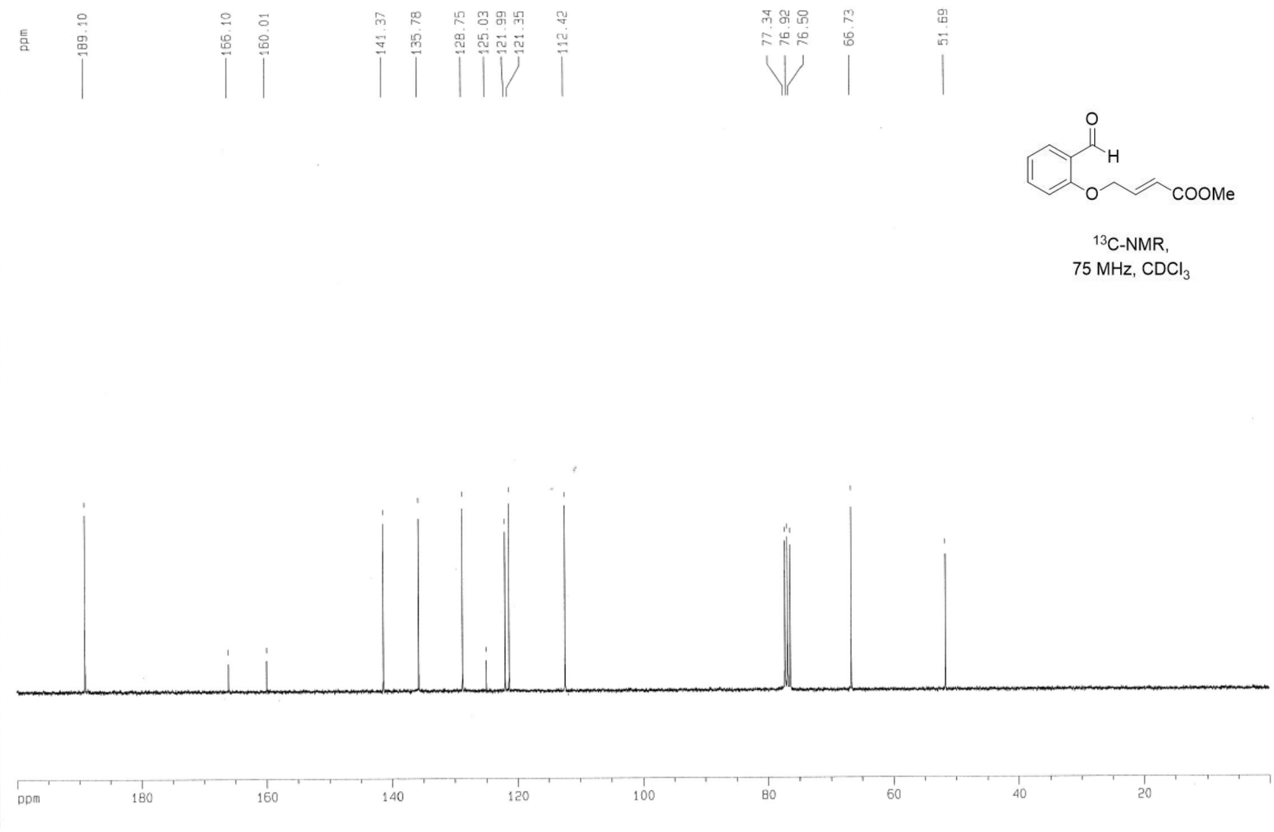
ppm
189.10
166.10
160.01
141.37
135.78
128.75
125.03
121.99
121.35
112.42
77.34
76.92
76.50
66.73
51.69
O
H
O
COOMe
$^{13}$C-NMR,
75 MHz, CDCl$_3$
ppm 180 160 140 120 100 80 60 40 20

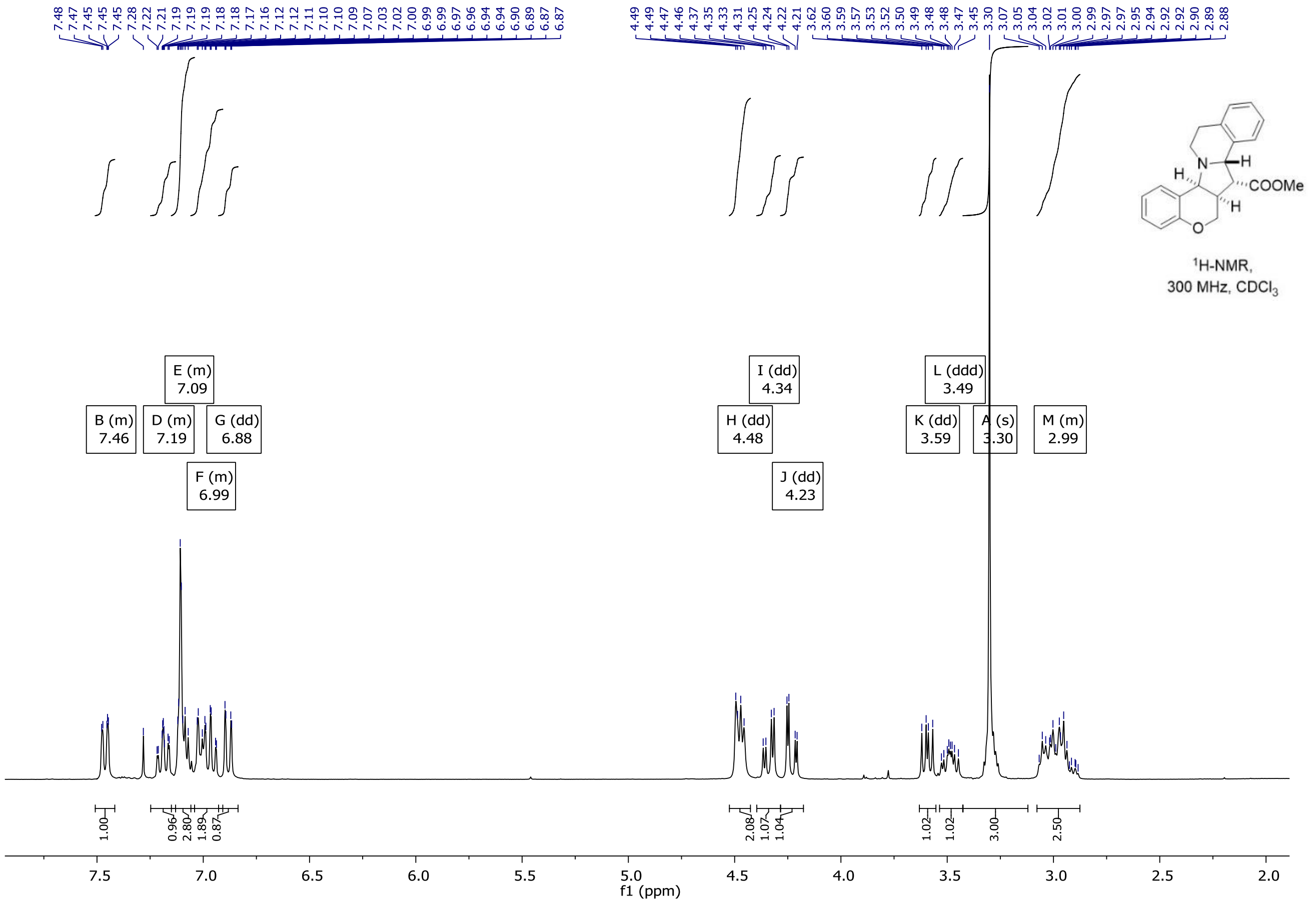
7.48 7.47 7.45 7.45 7.45 7.28 7.22 7.21 7.19 7.19 7.19 7.18 7.17 7.16 7.12 7.12 7.11 7.10 7.10 7.09 7.07 7.03 7.02 7.00 6.99 6.99 6.97 6.96 6.94 6.94 6.90 6.89 6.87 6.87
4.49 4.49 4.47 4.46 4.37 4.35 4.33 4.31 4.25 4.24 4.22 4.21 3.62 3.60 3.59 3.57 3.53 3.52 3.50 3.49 3.48 3.48 3.47 3.45 3.30 3.07 3.05 3.04 3.02 3.01 3.00 2.99 2.97 2.97 2.95 2.94 2.92 2.92 2.90 2.89 2.88
H
N
H
COOMe
H
O
1H-NMR,
300 MHz, CDCl3
E (m) 7.09
B (m) 7.46
D (m) 7.19
G (dd) 6.88
F (m) 6.99
I (dd) 4.34
H (dd) 4.48
J (dd) 4.23
L (ddd) 3.49
K (dd) 3.59
A (s) 3.30
M (m) 2.99
1.00
0.96
2.80
1.89
0.87
2.08
1.07
1.04
1.02
1.02
3.00
2.50
7.5 7.0 6.5 6.0 5.5 5.0 4.5 4.0 3.5 3.0 2.5 2.0
f1 (ppm)

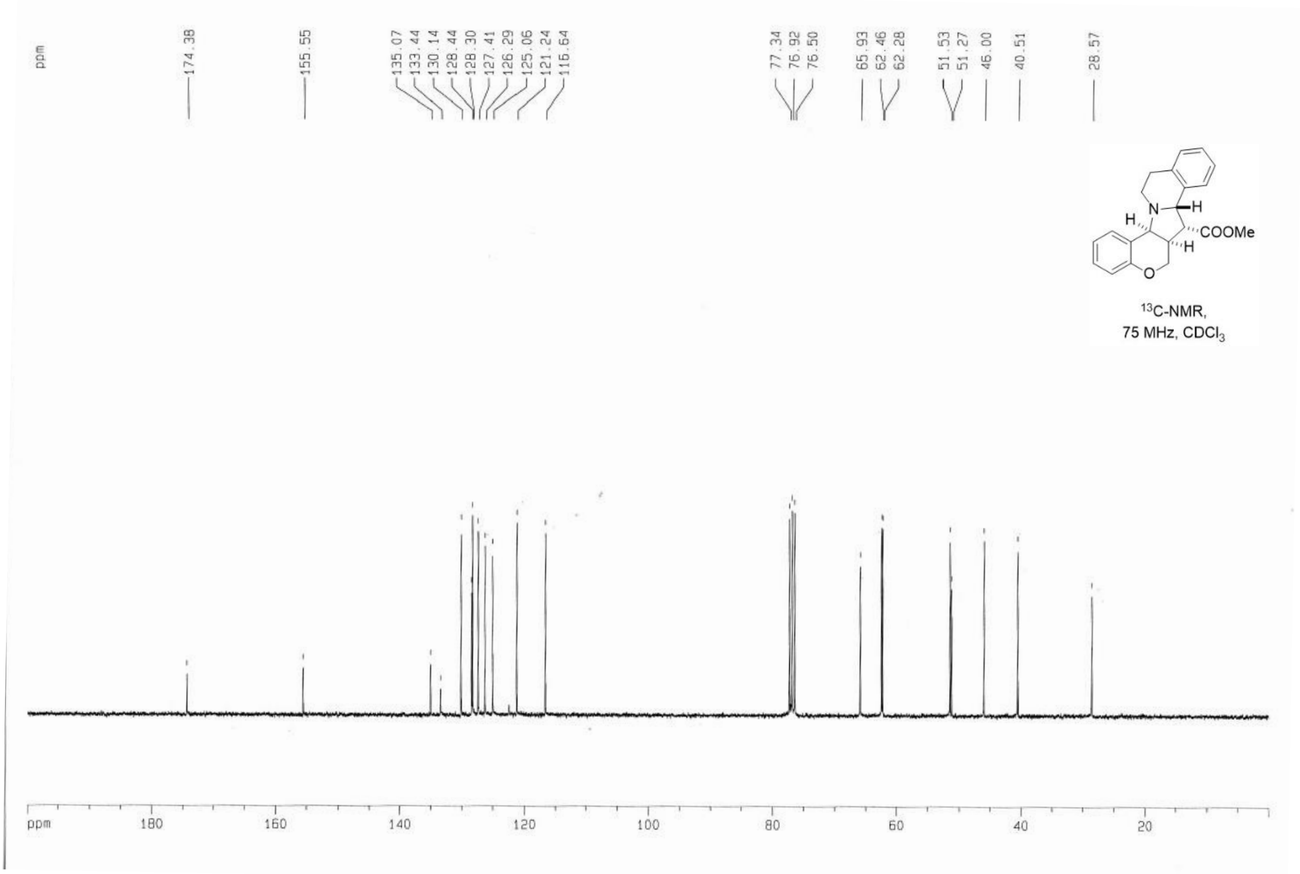
ppm
174.38
155.55
135.07 133.44 130.14 128.44 128.30 127.41 126.29 125.06 121.24 116.64
77.34 76.92 76.50
65.93 62.46 62.28
51.53 51.27 46.00 40.51
28.57
H
N
H
COOMe
H
O
13C-NMR,
75 MHz, CDCl3
ppm 180 160 140 120 100 80 60 40 20

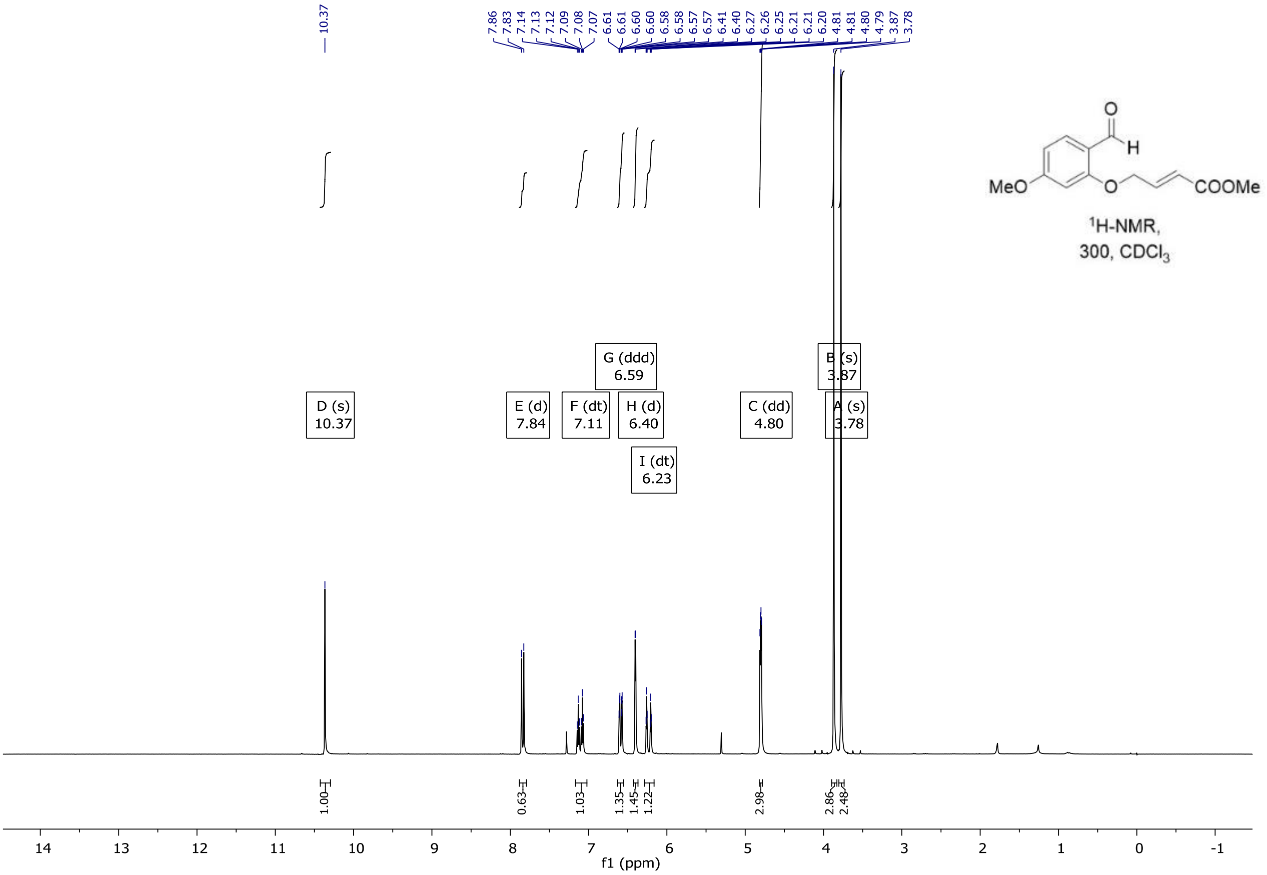
10.37
7.86 7.83 7.14 7.13 7.12 7.09 7.08 7.07 6.61 6.61 6.60 6.60 6.58 6.58 6.57 6.57 6.41 6.40 6.27 6.26 6.25 6.21 6.21 6.20 4.81 4.81 4.80 4.79 3.87 3.78
MeO
O
H
COOMe
$^1$H-NMR,
300, CDCl$_3$
G (ddd) 6.59
B (s) 3.87
D (s) 10.37
E (d) 7.84
F (dt) 7.11
H (d) 6.40
C (dd) 4.80
A (s) 3.78
I (dt) 6.23
1.00
0.63
1.03
1.35
1.45
1.22
2.98
2.86
2.48
14 13 12 11 10 9 8 7 6 5 4 3 2 1 0 -1
f1 (ppm)

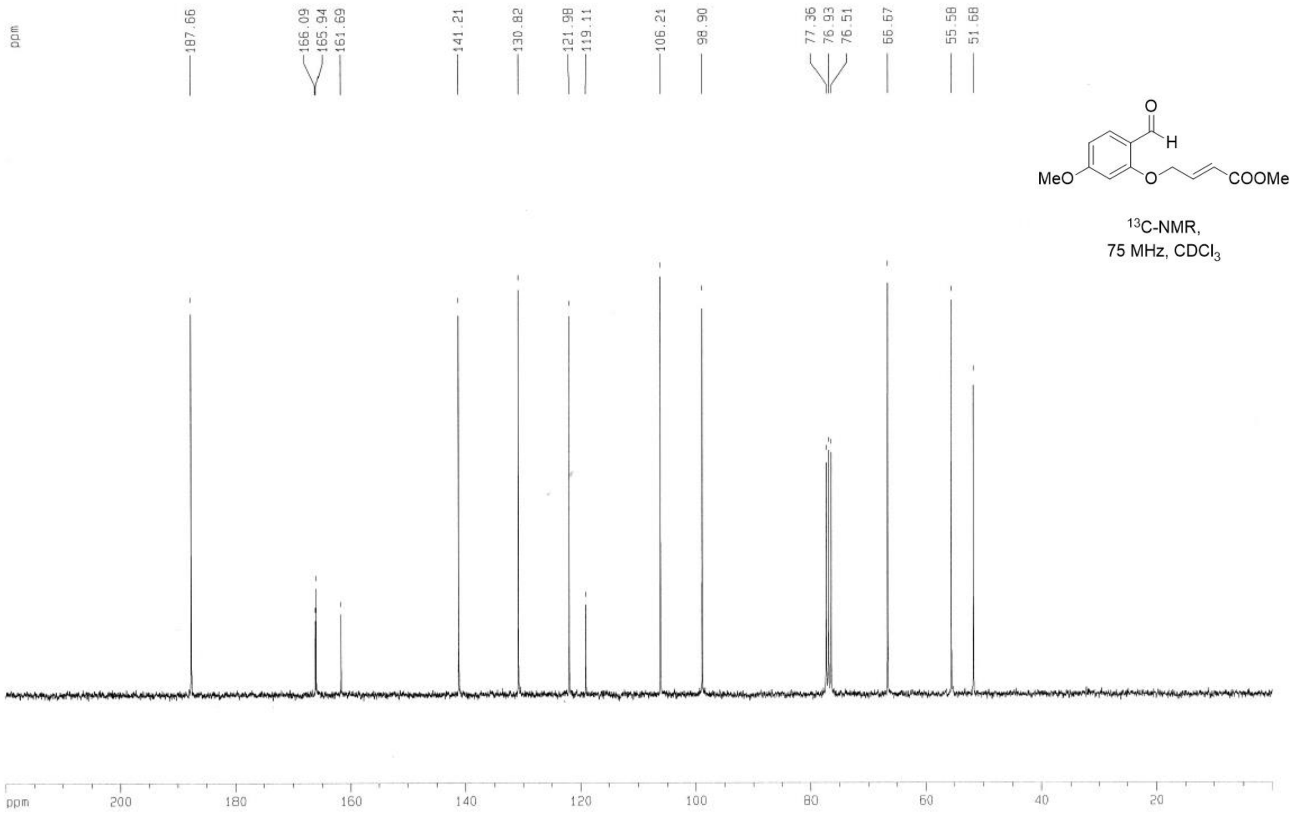
ppm
187.66
166.09 165.94 161.69
141.21
130.82
121.98 119.11
106.21
98.90
77.36 76.93 76.51
66.67
55.58 51.68
MeO
O
H
COOMe
$^{13}$C-NMR,
75 MHz, CDCl$_3$
ppm 200 180 160 140 120 100 80 60 40 20

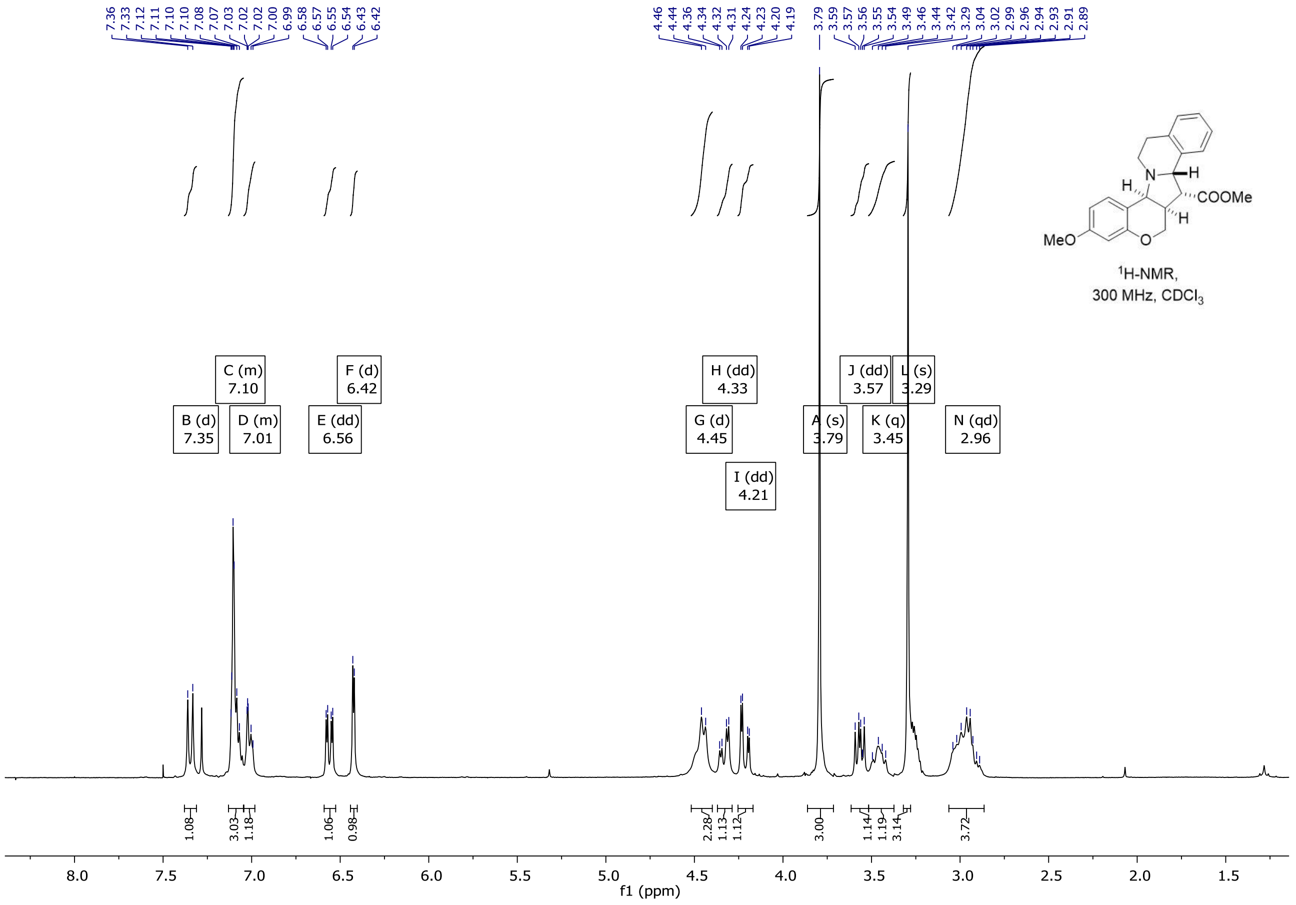
7.36 7.33 7.12 7.11 7.10 7.10 7.08 7.07 7.03 7.02 7.02 7.00 6.99 6.58 6.57 6.55 6.54 6.43 6.42
4.46 4.44 4.36 4.34 4.32 4.31 4.24 4.23 4.20 4.19 3.79 3.59 3.57 3.56 3.55 3.54 3.49 3.46 3.44 3.42 3.29 3.04 3.02 2.99 2.96 2.94 2.93 2.91 2.89
H
COOMe
H
MeO
O
N
H
$^1$H-NMR,
300 MHz, $CDCl_3$
C (m) 7.10
F (d) 6.42
H (dd) 4.33
J (dd) 3.57
L (s) 3.29
B (d) 7.35
D (m) 7.01
E (dd) 6.56
G (d) 4.45
A (s) 3.79
K (q) 3.45
N (qd) 2.96
I (dd) 4.21
1.08 3.03 1.18 1.06 0.98 2.28 1.13 1.12 3.00 1.14 1.19 3.14 3.72
8.0 7.5 7.0 6.5 6.0 5.5 5.0 4.5 4.0 3.5 3.0 2.5 2.0 1.5
f1 (ppm)

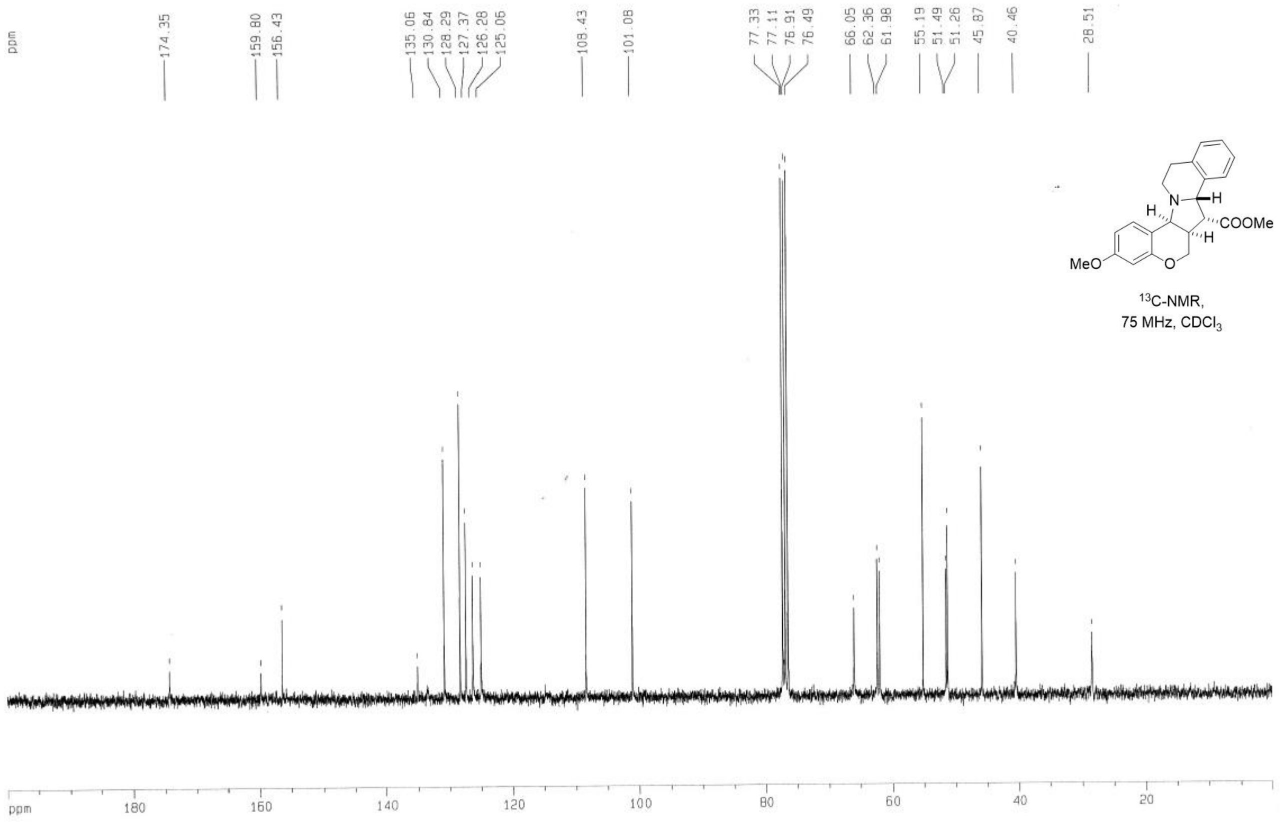
ppm
174.35 159.80 156.43 135.06 130.84 128.29 127.37 126.28 125.06 108.43 101.08 77.33 77.11 76.91 76.49 66.05 62.36 61.98 55.19 51.49 51.26 45.87 40.46 28.51
H
COOMe
H
MeO
O
N
H
$^{13}$C-NMR,
75 MHz, $CDCl_3$
ppm 180 160 140 120 100 80 60 40 20

## 2. UV-Visible spectra of compounds PA1, PA2, 1 and 4a.

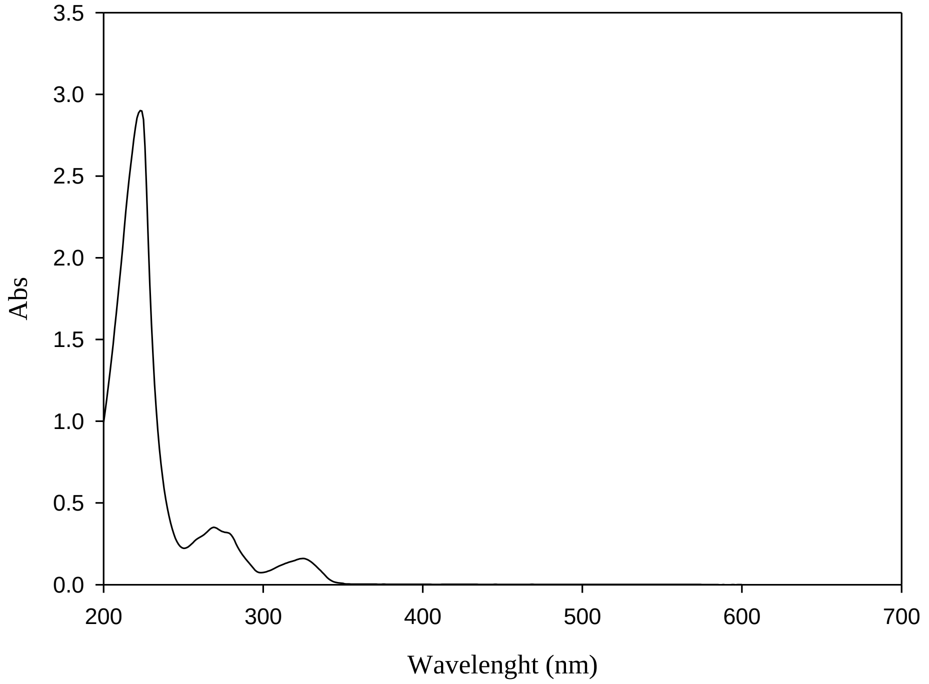


**Figure S1.** UV-Visible spectra of a solution $10^{-4}$ M of 2-Naphtol (**PA1**) in Acetonitrile.

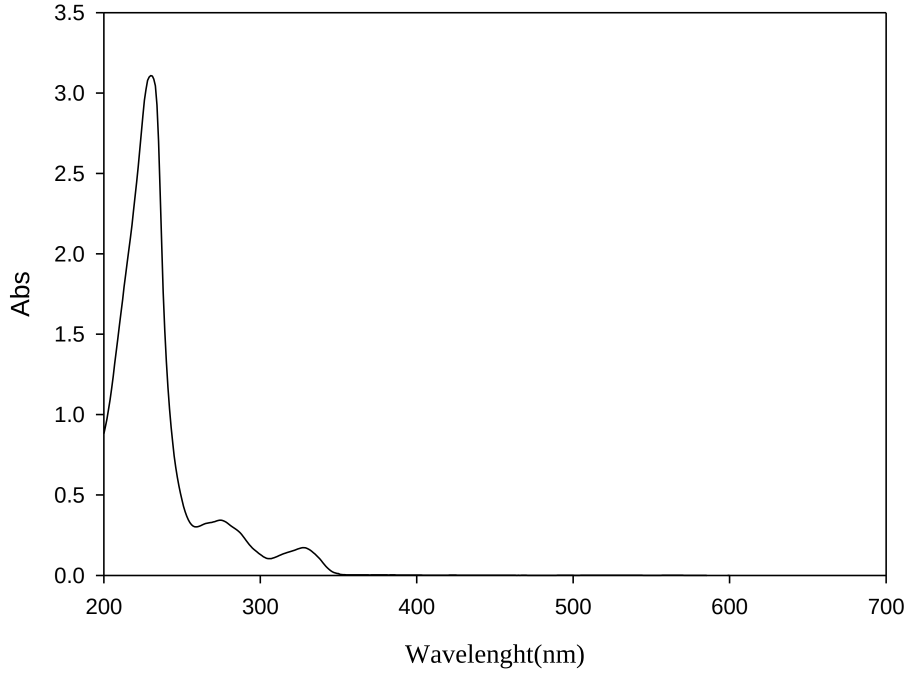


**Figure S2**. UV-Visible spectra of a solution $10^{-4}$ M of 7-Bromo-2-naphtol in Acetonitrile

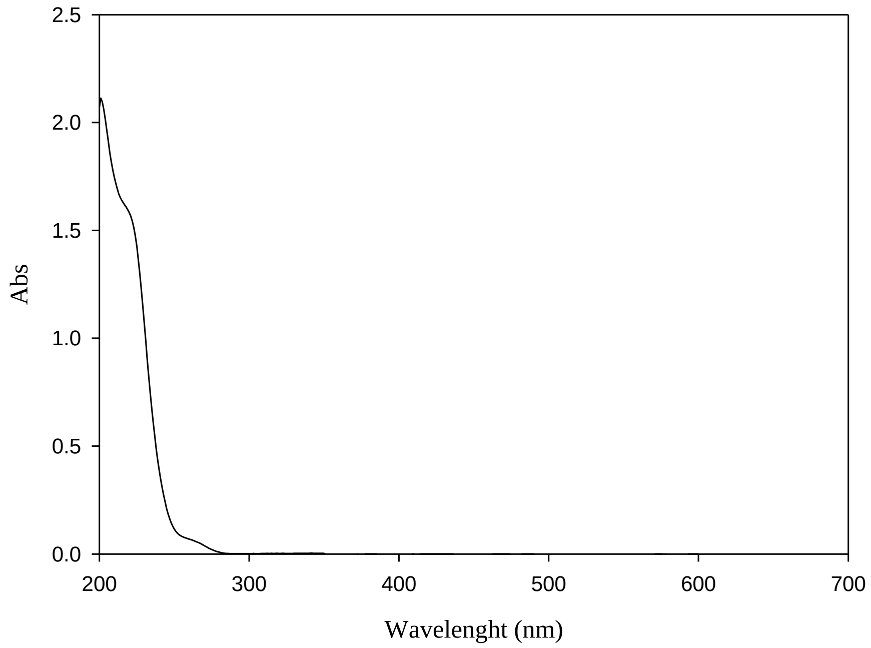


**Figure S3**. UV-Visible spectra of a solution $10^{-4}$ M of Benzyl 2,2,2-trichloroacetimidate **1** in Acetonitrile

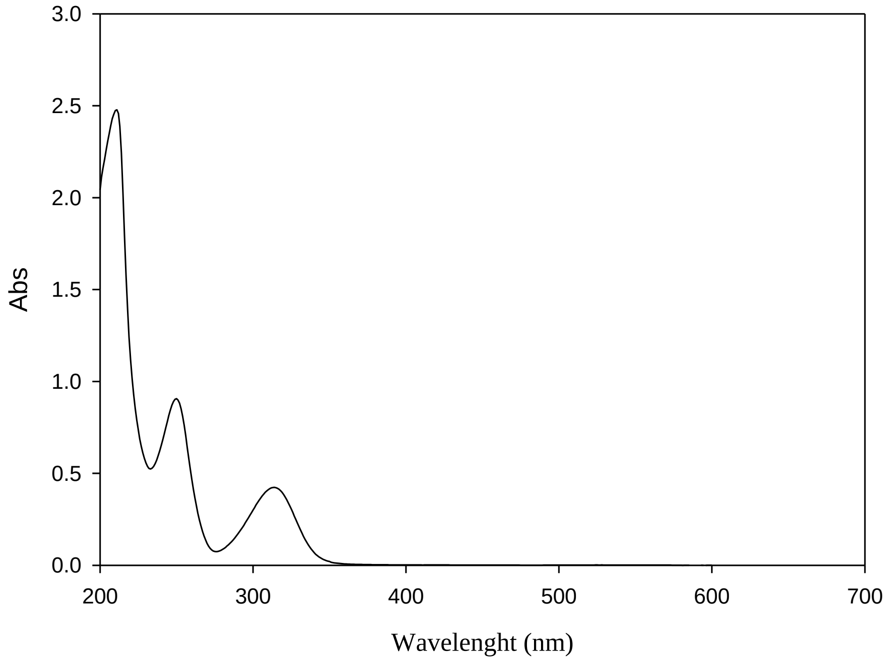


**Figure S4**. UV-Visible spectra of a solution $10^{-4}$ M of (E)-methyl 4-(2-formylphenoxy)but-2-enoate **4a** in Acetonitrile

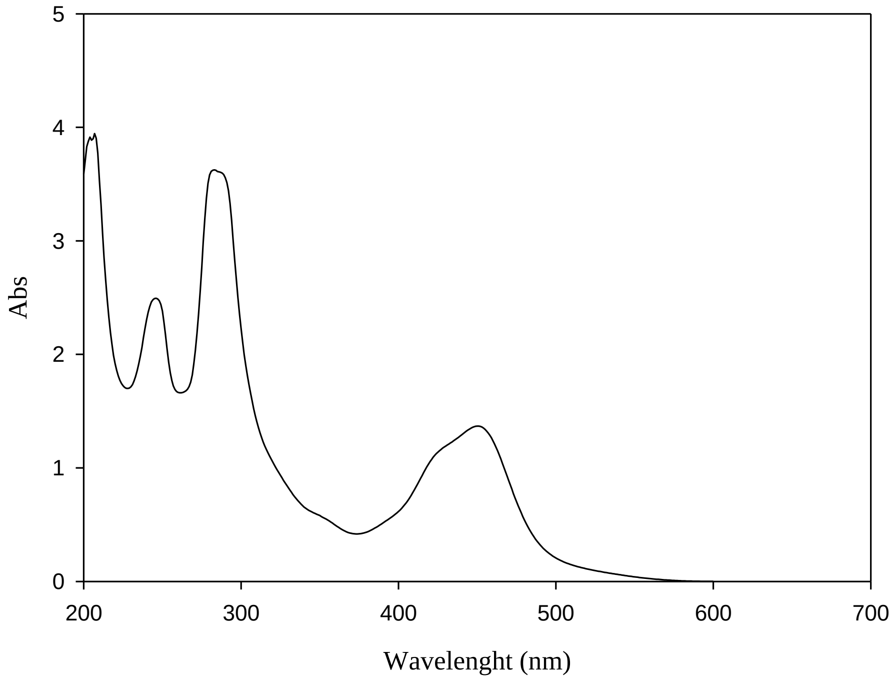


**Figure S5**. UV-Visible spectra of a solution $10^{-4}$ M of Tris(2,2'-bipyrydyl)dichlororuthenium(II) hexahydrate in Acetonitrile
